# Integrating Tumour Biology into Surgical Decision-Making for Metastatic Colorectal Cancer: A Real-World Cohort Analysis Adapted for Clinical Colorectal Cancer

**DOI:** 10.3390/cancers18193070

**Published:** 2026-09-22

**Authors:** Mariana Paula Cunha Gonçalves, Pedro Henrique Faria Silva Trocoli-Couto, Augusto Henrique Marchiodi, Lucas Toledo Bernardo de Carvalho, Yuiti Pedro Henrique Yamashita, Henrique Araújo Lima, Vivian Resende

**Affiliations:** 1 Department of Surgery, School of Medicine, Federal University of Minas Gerais, Belo Horizonte 30130-100, MG, Brazilaugustomarchiodi@ufmg.br (A.H.M.);; 2 Thoracic and Gastrointestinal Oncology, Grupo Orizonti, Belo Horizonte 30210-080, MG, Brazil

**Keywords:** metastatic colorectal cancer, metastasectomy, tumour sidedness, RAS mutation, overall survival

## Abstract

Background: Metastatic colorectal cancer (mCRC) is biologically heterogeneous, resulting in variable outcomes despite similar disease burden and treatment. Although metastasectomy offers the only potentially curative option for selected patients, surgical selection remains largely anatomy based. Whether biological factors continue to influence outcomes after resection remains unclear. Methods: We retrospectively analysed 290 consecutive patients with stage IV colorectal adenocarcinoma treated at a Brazilian tertiary oncology centre (2003–2019). Tumour sidedness, RAS mutational status, and metastasectomy were evaluated for overall survival (OS) using Kaplan–Meier analysis and multivariable Cox regression in the overall cohort and the metastasectomy subgroup. Results: Median OS was 40.2 months. Patients undergoing metastasectomy had significantly longer survival than those managed non-operatively (median 116.0 vs. 30.1 months; 5-year OS 63% vs. 14%; *p* < 0.001). The following variables remained independently associated with improved survival: metastasectomy (HR 4.58, 95% CI 2.82–7.44; *p* < 0.001), RAS status (wild type vs. mutated: HR 2.77, 95% CI 1.65–4.64; *p* < 0.001), age (>70 ys vs. ≤ 70 HR 0.58, 95% CI 0.37–0.92; *p* = 0.021) and metastatic presentation (synchronous vs. metachronous: HR 0.61 95% CI 0.39–0.95; *p* = 0.028). RAS status remained associated with survival in patients undergoing metastasectomy (HR 3.75, 95% CI 1.46–9.60; *p* = 0.006). Conclusions: Tumour biology remains a dominant determinant of survival in mCRC even after metastasectomy. These data challenge purely anatomy-based surgical paradigms and support integrating molecular and clinicopathological risk into post-resection management and treatment planning.

## Simple Summary

Metastatic colorectal cancer can behave very differently from one patient to another, even when the amount of disease appears similar. Surgery to remove metastatic tumours can offer long-term survival for carefully selected patients, but decisions about surgery are often based mainly on whether the tumours can be technically removed. This study investigated whether characteristics of the cancer itself, particularly genetic changes and the location of the original tumour in the colon or rectum, also influence survival after surgery. We analysed 290 patients with metastatic colorectal cancer treated at a Brazilian cancer centre and compared their outcomes according to treatment and tumour characteristics. We found that patients who underwent surgery had substantially longer survival; however, among patients who underwent surgery, those with RAS gene alterations had poorer outcomes. These findings suggest that tumour biology should be considered alongside anatomical factors when selecting patients for metastatic surgery.

## 1. Introduction

Metastatic colorectal cancer (mCRC) is a paradigmatic example of the widening gap between therapeutic sophistication and the conceptual frameworks that guide clinical decision-making. Over two decades, combination chemotherapy, biologically targeted agents, and molecular stratification have produced meaningful survival gains. Yet these advances have simultaneously exposed a fundamental limitation: patients with ostensibly similar disease burden and treatment exposure continue to follow markedly divergent trajectories [1,2,3].

This heterogeneity cannot be explained by anatomical staging alone; it reflects the biological diversity of colorectal cancer, now understood as a spectrum of molecularly distinct entities rather than a single disease. Large-scale genomic and transcriptomic analyses have defined discrete consensus molecular subtypes with characteristic patterns of oncogenic signalling, immune microenvironment composition and metastatic behaviour [4,5,6]. These differences translate directly into variability in treatment response, recurrence patterns and long-term survival.

Among the most clinically actionable determinants are RAS mutational status and primary tumour sidedness. Activating RAS mutations confer resistance to anti-EGFR therapy and a more aggressive phenotype, with greater metastatic competence and shorter survival across treatment settings [7,8,9]. In parallel, sidedness has emerged as an accessible surrogate for deeper biological divergence: right-sided tumours are enriched for BRAF mutation, microsatellite instability, the CpG-island methylator phenotype and distinct stromal–immune features, all contributing to inferior prognosis and differential therapeutic responsiveness [10,11,12,13,14].

Despite growing incorporation of these determinants into systemic algorithms, surgical decision-making for mCRC has remained anchored in anatomy. Metastasectomy is the only potentially curative intervention in selected patients, particularly in oligometastatic disease, and is associated with long-term survival in carefully selected cohorts [15,16,17]. However, prevailing definitions of resectability rest on technical feasibility and disease distribution, with limited formal integration of molecular risk.

This disconnect is increasingly difficult to justify. CALGB/SWOG 80405 and FIRE-3 showed that RAS status and sidedness are not merely prognostic but active determinants of therapeutic benefit [18,19,20]; PARADIGM prospectively confirmed biologically conditioned first-line benefit in RAS wild-type, left-sided tumours [21]; and CAIRO5 demonstrated that biologically guided systemic strategies modulate the probability of achieving secondary resectability—implying that surgical candidacy is a dynamic, biologically influenced state rather than a fixed anatomical one [22].

An unresolved question follows directly: to what extent does tumour biology continue to shape outcomes after apparently complete resection of metastatic disease? Does metastasectomy neutralise adverse biology, or does intrinsic tumour genotype persist as a dominant prognostic force even after optimal local control? We addressed this by evaluating the combined impact of sidedness, RAS status and metastasectomy on OS in a real-world mCRC cohort, integrating anatomical and biological variables within a single analytical framework to inform a more precise, clinically meaningful definition of resectability.

## 2. Materials and Methods

### 2.1. Study Design and Population

This retrospective observational cohort included consecutive adults with histologically confirmed stage IV colorectal adenocarcinoma treated between January 2003 and December 2019 at a tertiary oncology referral centre in Belo Horizonte, Brazil. The local research ethics committee approved the original study under protocol number 4.068.436 on 6 June 2020. Patients were identified from the institutional oncology registry and followed until death or closure of the database.

Three hundred patients met these criteria. Ten were excluded because the location of the primary tumour could not be classified as right- or left-sided. This was a defining variable of the study, leaving 290 evaluable patients: 121 who underwent metastasectomy and 169 managed non-operatively with systemic therapy, best supportive care, or both. Eligibility was independent of the site of metastatic involvement, of the timing of metastatic presentation and of the systemic regimen received; the cohort was therefore not confined to colorectal liver metastases.

### 2.2. Variables and Definitions

Variables selected for analysis included demographic, clinical, pathological, molecular and treatment characteristics, such as age at diagnosis, sex, ECOG performance status, comorbidity, histological subtype, differentiation grade, pathological T and N stage (when the primary had been resected), primary tumour location, timing of metastatic presentation (synchronous or metachronous), metastasectomy status, site of resected metastatic disease, KRAS/NRAS status and exposure to complete systemic therapy. Primary tumour sidedness followed the original dataset: tumours from the caecum, ascending colon, and transverse colon up to the splenic flexure were classified as right-sided, and those from the descending colon, sigmoid colon, and rectum were grouped as left-sided for the principal survival comparisons. KRAS and NRAS testing was performed in routine practice on available tumour samples. As a real-world cohort spanning several therapeutic eras, some biomarkers now considered standard (e.g., BRAF, microsatellite instability) were not systematically available.

Metastatic presentation was classified as synchronous when distant disease was documented at or within six months of the diagnosis of the primary tumour, and metachronous thereafter. Margin status was recorded as R0 for complete resection with microscopically negative margins and R1 for microscopically involved margins. The extent of hepatectomy was classified as major for resection of three or more contiguous Couinaud segments and minor otherwise, and the sequencing of resection of the primary tumour and its hepatic metastases as simultaneous or staged. The interval from the diagnosis of metastatic disease to metastasectomy was computed from the documented operative dates.

Metastasectomy was defined as surgical resection performed with curative intent of all radiologically identified metastatic disease, irrespective of anatomical site. Diagnostic biopsy, palliative debulking and procedures undertaken for symptom control without the intention of complete clearance were not classified as metastasectomy.

Selection for surgery was determined prospectively within a multidisciplinary tumour board comprising medical oncology, surgical oncology, hepatobiliary and thoracic surgery, radiology and pathology. No protocol-defined algorithm governed the decision, and assignment was not randomised. Resectability was defined primarily in technical and oncological terms: the anticipated ability to achieve complete macroscopic and microscopic clearance of all radiologically identified disease, with preservation of an adequate functional remnant of the involved organ, in a patient whose performance status and organ reserve permitted the planned procedure. For hepatic resection, this required a remnant retaining intact vascular inflow, venous outflow and biliary drainage, as well as sufficient predicted volume; for pulmonary resection, it required sufficient predicted post-operative respiratory function.

Extrahepatic metastatic disease was not treated as an absolute contraindication but was assessed in the same manner, namely whether all identified sites could be cleared. Disease that was diffuse, not resectable in its entirety, or radiologically progressive under systemic therapy was regarded as a contraindication. Neither RAS mutational status nor primary tumour sidedness was used as a criterion for or against surgery at any point during the study period, which permits these variables to be examined as prognostic markers within the operated group without circularity.

### 2.3. Metastatic Disease Burden

For patients undergoing hepatic resection, indicators of metastatic disease burden were abstracted: lobar distribution, number of hepatic nodules, diameter of the largest nodule, and pre-operative carcinoembryonic antigen concentration.

### 2.4. Systemic Therapy

Palliative systemic regimens were recorded individually for the first and second lines rather than as a composite category, and classified as fluoropyrimidine–oxaliplatin based, irinotecan-based, single-agent fluoropyrimidine, or combined with an anti-EGFR antibody. Anti-EGFR agents comprised cetuximab and panitumumab and were administered to patients with RAS wild-type disease in accordance with the clinical practice and reimbursement policy in force at the time. Bevacizumab was not reimbursed for metastatic colorectal cancer within the Brazilian public health system during most of the study period and was not administered in this cohort. For patients undergoing hepatic resection, the regimen administered before surgery and the intent of systemic therapy—adjuvant, neoadjuvant or conversion—were recorded separately.

The number of cycles administered was not recorded systematically across the full study period. Completion of first-line therapy was therefore defined operationally as administration of the intended number of cycles of the planned regimen without premature discontinuation for toxicity, disease progression or clinical deterioration, and was ascertained at the conclusion of first-line therapy rather than at baseline. Because this variable can only be observed in patients who survive and remain clinically fit long enough to receive treatment, it was additionally modelled as a time-dependent covariate.

### 2.5. Treatment Era

Because recruitment spanned sixteen years, encompassing the consolidation of oxaliplatin- and irinotecan-based doublets, the introduction of anti-EGFR antibodies and their subsequent restriction to RAS wild-type disease, treatment era was defined a priori as a covariate and categorised according to the availability of routine molecular characterisation and of targeted agents within the institution. A sensitivity analysis restricted to the contemporary era is reported alongside the primary analysis. The full cohort was retained as the primary analysis, since restriction would substantially reduce statistical power in a single-institution series and compromise the real-world character of the study.

### 2.6. Statistical Analysis

Baseline characteristics are reported as counts and percentages, computed among patients in whom the variable was documented, and were compared between treatment groups using the chi-square or Fisher’s exact test as appropriate; age was compared using the Mann–Whitney U test.

The primary endpoint was overall survival, defined as the interval from diagnosis of metastatic disease to death from any cause. Survival was estimated by the Kaplan–Meier method and compared by log-rank testing, and median follow-up was estimated by the reverse Kaplan–Meier method. All survival estimates and curves were computed from individual patient-level time-to-event data, and the numbers at risk displayed beneath each curve derive from the same dataset.

Cox proportional-hazards models estimated hazard ratios (HRs) with 95% confidence intervals (CIs); models were fitted both for the full cohort and, in a pre-specified subgroup, restricted to patients undergoing metastasectomy. The proportional hazards assumption was examined by inspection of scaled Schoenfeld residuals. Given the number of deaths observed within the metastasectomy subgroup, the number of covariates entered into models restricted to it was deliberately constrained to avoid overfitting.

Because metastasectomy necessarily occurs after the diagnosis of metastatic disease, classifying it as a fixed baseline characteristic would attribute to surgery the survival time that patients were required to accrue to reach it [22]. Two complementary strategies were therefore applied. First, metastasectomy was modelled as a time-dependent covariate, with the dataset restructured into a counting process format in which each operated patient contributes unexposed person-time from diagnosis until the date of surgery and exposed person-time thereafter. Second, landmark analyses were performed at three, six and twelve months, restricted at each landmark to patients alive at that time and defining exposure status as at the landmark [23]. Both sets of estimates are reported, and the time-dependent estimate is presented as the primary estimate of the association between metastasectomy and survival. Completion of systemic therapy was re-specified within the same framework.

Recurrence-free survival was analysed among patients undergoing hepatic metastasectomy, measured from the date of surgery to the first radiologically or histologically confirmed recurrence at any site or death, whichever occurred first.

Because of the retrospective design, associations involving metastasectomy were interpreted cautiously, acknowledging selection bias. No causal inference methods were applied. The analyses establish whether biological covariates retain independent prognostic significance among operated patients; they do not test whether these characteristics modify the relative benefit conferred by surgery, an inference that would require formal interaction testing within a design capable of estimating the counterfactual outcome in comparable unresected patients. The biological stratification reported here should be regarded as hypothesis generating rather than as a validated prognostic tool. Two-sided *p* values below 0.05 were considered statistically significant; analyses were performed in R (version 4.6.1).

## 3. Results

### 3.1. Cohort and Survival Overview

A total of 290 patients were included, of whom 121 underwent metastasectomy, and 169 were managed non-operatively. Median follow-up was 57.3 months, and 162 deaths were observed. Median OS for the entire cohort was 40.2 months (95% CI 32.0–49.4). Survival probabilities at 1,2,3,4, and 5 years were 85%, 68%, 52%, 46% and 37%, respectively.

Patients selected for metastasectomy differed systematically from those managed non-operatively. They were younger (median 58 vs. 64 years; *p* = 0.001), had better performance status (ECOG 0 in 79.3% vs. 63.3%, with no operated patient having ECOG 2 or worse; *p* = 0.003), more frequently presented with metachronous disease (57.9% vs. 34.9%; *p* < 0.001), and were more completely genotyped (RAS status determinable in 90.9% vs. 61.5%; *p* < 0.001). Neither primary tumour location (right-sided in 28.9% vs. 37.9%; *p* = 0.145) nor RAS status among genotyped patients (mutated in 58.2% vs. 68.3%; *p* = 0.166) differed significantly between the groups, indicating that the two biological characteristics examined here were not themselves determinants of surgical selection. Baseline characteristics are summarised in Table 1.

### 3.2. Distribution and Burden of Metastatic Disease

Among patients undergoing metastasectomy, the resected site was hepatic in 51.2%, pulmonary in 18.2%, peritoneal in 16.5%, nodal in 4.1%, ovarian in 3.3%, and other or combined in 6.6%. Extrahepatic resections therefore accounted for approximately half of all procedures, and peritoneal, nodal and ovarian disease together for approximately one-quarter.

Within the hepatic resection subgroup, disease was unilobar in 74.0% and bilobar in 26.0%. Hepatic involvement was solitary in 58.9% and comprised four or more nodules in 17.8%. The median was one nodule (interquartile range 1–3), and the largest nodule was 40 mm (interquartile range 20–50). Pre-operative carcinoembryonic antigen, available in 94.5%, had a median of 13.0 ng/mL (interquartile range 3.0–89.0) and exceeded 5 ng/mL in 68.1%. Major hepatectomy was performed in 45.2% and minor resection in 54.8%. Resection of the primary tumour and hepatic metastases was performed simultaneously in 11.0% and in a staged manner in 89.0%. Histopathological margins were free of tumours in 78.1%. The median interval from diagnosis of metastatic disease to metastasectomy was 216 days (interquartile range 85–323), exceeding 90 days in 72.9% of patients and 180 days in 57.1%.

### 3.3. Systemic Treatment

First-line palliative therapy was fluoropyrimidine–oxaliplatin based in 78.5% of patients who underwent metastasectomy and 76.9% of those managed non-operatively. Irinotecan-based therapy was used in in 9.1% and 8.9%, single-agent fluoropyrimidine in 9.9% and 11.2%, and combined with an anti-EGFR antibody in 1.7% and 3.0%. A second line was received by 57.9% and 56.2%, respectively, and most often irinotecan based. No component of first- or second-line treatment differed significantly between the groups, and total anti-EGFR exposure across both lines was 5.0% and 4.7% (*p* = 1.00).

Among patients undergoing hepatic resection, pre-operative systemic therapy was administered in 89.0%, predominantly as FLOX in 75.3%, with 8.2% receiving fluorouracil and leucovorin alone; 11.0% proceeded directly to surgery. Where the intent of treatment was documented, it was adjuvant in 64.4%, neoadjuvant in 11.1% and given with conversion intent in 5.6%. First-line therapy was completed, as defined in the Methods, in 33.1% of operated and 42.0% of non-operated patients (*p* = 0.154).

### 3.4. Metastasectomy and the Survival Gradient

The strongest survival gradient was observed by metastasectomy status. Patients undergoing resection of metastatic disease had a median OS of 116.0 months versus 30.1 months among those managed non-operatively, with 5-year OS of 63% and 14%, respectively (Figure 1). Although striking, this difference must be read in the context of selection bias, since surgical candidates are also more likely to have favourable biology, better performance status and lower disease burden.

A second consideration applies to the magnitude of the estimate. Because surgery followed the diagnosis of metastatic disease by a median of more than seven months, the conventional specification attributes to the operation a period of survival that operated patients were required to accrue in order to undergo it. In the conventional multivariable model the absence of metastasectomy carried the largest adjusted hazard (HR 4.58; 95% CI 2.82–7.44; *p* < 0.001; Table 2). When metastasectomy was modelled as a time-dependent covariate, this attenuated to 3.54 (95% CI 2.17–5.77; *p* < 0.001), and landmark analyses at three, six and twelve months yielded 2.30 (1.39–3.81), 2.50 (1.53–4.09) and 3.14 (1.87–5.26), respectively (Supplemental Table S1). The association remained large and highly significant under every specification.

### 3.5. Tumour Biology Across the Cohort

Tumour biology remained highly relevant. In multivariable analysis (Table 2), RAS mutation was independently associated with inferior survival (HR 2.77; 95% CI 1.65–4.64; *p* < 0.001), an estimate that was essentially invariant across the specifications used to correct for the timing of surgery, ranging from 2.73 to 2.96 and remaining significant in all of them (Supplemental Table S1). Right-sided primaries were associated with worse outcome than left-sided tumours on unadjusted analysis (median OS 30.0 vs. 50.2 months; log-rank *p* = 0.001; HR 1.69; 95% CI 1.23–2.32); the estimate attenuated to 1.56 (1.01–2.40) when adjusted for RAS status and metastasectomy alone and to 1.44 (0.93–2.23; *p* = 0.100) in the fully adjusted model. Younger age (HR 0.58; 0.37–0.92; *p* = 0.021) and metachronous presentation (HR 0.61; 0.39–0.95; *p* = 0.028) were independently associated with better survival.

Completion of first-line systemic therapy showed no independent association with survival, either in the conventional specification (HR 1.22; 95% CI 0.80–1.88; *p* = 0.357) or when modelled as a time-dependent covariate (HR 1.25; 0.81–1.92; *p* = 0.323; Supplemental Table S1). Therefore, it is not reported as a prognostic factor.

### 3.6. Biology Persisted Within the Resected Subgroup

Among patients undergoing metastasectomy with curative intent, biology continued to matter (Table 3). RAS mutation remained independently associated with worse OS (HR 3.75; 95% CI 1.46–9.60; *p* = 0.006; unadjusted HR 5.52; 2.25–13.51), as did pN2 nodal status in the resected primary specimen (HR for pN0–1 vs. pN2 0.38; 95% CI 0.18–0.77; *p* = 0.008). Five-year OS after metastasectomy was 86.2% in RAS wild-type and 50.8% in RAS-mutant disease; median OS was not reached in the former group and was 80.4 months in the latter (log-rank *p* < 0.001; Figure 2A).

Right-sided location was likewise associated with markedly shorter survival within this subgroup on unadjusted analysis (median OS 55.6 vs. 148.2 months; log-rank *p* = 0.016; HR 2.15; 95% CI 1.14–4.05), but did not retain independent significance after adjustment for RAS status (HR 1.47; 0.72–2.99; *p* = 0.292). Sidedness and RAS status were correlated in this cohort: among operated patients, RAS mutation was present in 71.9% of right-sided and 52.6% of left-sided tumours.

The persistence of these biological hazards in both the full cohort and the surgically resected subgroup is depicted in Figure 2 and supports the interpretation that long-term outcomes after metastasectomy are not determined by technical resectability alone.

### 3.7. Recurrence After Hepatic Metastasectomy

Recurrence occurred in 70.0% of patients undergoing hepatic resection. Median recurrence-free survival was 15.0 months, with 64.7% of patients free of recurrence at one year and 20.9% at three years. Among those who recurred and in whom the site was documented, the first site of recurrence was hepatic alone in 34.0%, extrahepatic alone in 30.0% and both in 18.0%.

## 4. Discussion

This study supports a critical reframing of decision-making for mCRC. While metastasectomy has long been positioned as the central curative strategy in selected patients, our findings indicate that its benefit is not an intrinsic property of resection itself but a conditional outcome heavily modulated by underlying tumour biology.

The magnitude of the survival difference between resected and non-operative patients is consistent with historical surgical series [14,15,16]. However, this apparent benefit reflects a complex interplay between treatment effect and biological selection. Because metastasectomy is a post-baseline exposure occurring at a median of more than seven months after the diagnosis of metastatic disease, part of the survival advantage observed under a conventional specification is guaranteed by construction. Correcting for this attenuated the estimate by approximately one-fifth under time-dependent modelling and by more under the landmark specifications, the association remained large and highly significant throughout is itself informative: the magnitude of surgical benefit in this cohort is not an artefact of how exposure was defined.

Selection operated alongside this. Patients eligible for metastasectomy were highly selected, characterised not only by limited anatomical burden but also by a more favourable clinical phenotype, an observation increasingly recognised yet insufficiently operationalised in practice, and quantified here in Table 1. What can be stated more specifically is that primary tumour location and RAS status did not differ between the groups, so the prognostic effects of these markers within the operated group are not attributable to the process by which patients were selected for surgery.

A central, clinically meaningful finding is the persistence of RAS mutational status as independent prognostic determinants even within the metastasectomy cohort. This directly challenges the paradigm in which complete macroscopic clearance is assumed to reset disease trajectory. Instead, our data support a model in which tumour-intrinsic biology exerts sustained influence on outcomes, irrespective of the apparent completeness of resection. The magnitude is considerable: five-year survival after resection differed by thirty-five absolute percentage points according to RAS status alone, and the estimate was unchanged by correction for immortal time bias since RAS status is fixed at diagnosis and cannot be affected by the timing of surgery.

Right-sidedness carried a significant survival disadvantage both across the cohort and within the resected subgroup, aligning with a substantial body of evidence [10,11,12,13], but its effect attenuated progressively with adjustment and did not remain independent once RAS status was included. We interpret this as biological rather than as a failure of the finding and consider it worth reporting explicitly because it is frequently obscured. Sidedness and RAS status were correlated in this cohort, mutations being present in 71.9% of right-sided and 52.6% of left-sided tumours among operated patients. A substantial part of the prognostic signal historically attributed to anatomical location is therefore carried by the molecular alteration with which location is associated. Sidedness is best understood as an accessible surrogate for an underlying molecular phenotype, of greatest value where genotyping is unavailable. With 35 deaths in the operated subgroup, these data cannot definitively separate two correlated variables, and we do not claim to have done so.

Mechanistically, this is plausible. Oncogenic activation of the RAS–RAF–MEK–ERK axis enhances proliferation, resistance to apoptosis and metastatic competence [7,8,24]. In the metastatic setting, these features may facilitate micrometastatic persistence and early recurrence after resection, attenuating the long-term benefit of surgery; RAS-mutant tumours show higher recurrence rates after hepatic resection and reduced survival [9,24,25]. The recurrence pattern observed here is consistent with this mechanism: recurrence occurred in 70.0% of patients undergoing hepatic resection despite curative intent and tumour-free margins in 78.1%, and extrahepatic or combined disease was the first site in nearly half of documented cases, indicating that the disease resected was frequently already systemic. The adverse impact of right-sided tumours reflects a broader molecular divergence—enrichment for BRAF mutation, microsatellite instability, the CpG-island methylator phenotype and distinct stromal–immune interactions [10,11,12,26]—that is functionally relevant rather than merely prognostic.

These implications sharpen when contextualised within randomised evidence. CALGB/SWOG 80405 and FIRE-3 established sidedness and RAS status as active determinants of anti-EGFR benefit [18,19,27]; PARADIGM prospectively validated the superiority of EGFR inhibition over VEGF-directed therapy in RAS wild-type, left-sided tumours [20]; and CAIRO5 extended the paradigm beyond systemic therapy by showing that biologically stratified strategies influence the probability of secondary resectability [21]. Our study adds a further layer: even after resectability has been achieved and surgery performed, biology continues to dictate outcome. This undermines the binary classification of patients as “resectable” or “unresectable” and supports a continuum model in which biological risk persists across all treatment phases.

A feature of this cohort strengthens that inference. Exposure to targeted agents was low throughout the study period and did not differ between groups: anti-EGFR antibodies reached 5.0% and 4.7% of operated and non-operated patients, bevacizumab was unavailable within the public health system for most of the period, and fluoropyrimidine–oxaliplatin doublets were the backbone in approximately three-quarters of both groups. In populations with high anti-EGFR penetration, the survival disadvantage of RAS-mutant disease is difficult to separate from the exclusion of these patients from an effective therapy, since RAS status simultaneously determines prognosis and eligibility for treatment. Here it does not, and the prognostic effect observed is attributable to the intrinsic behaviour of the tumour rather than to differential access to treatment.

Clinically, a paradigm centred on technical feasibility alone risks misalignment between the intent of surgery and its durable result. Our data do not support withholding metastasectomy from technically resectable patients on the basis of adverse tumour biology, and establishing that biology modifies the benefit of surgery would require a design capable of estimating the counterfactual outcome in comparable unresected patients. What the data do support is a refinement of what follows the operation: RAS-mutant patients undergoing metastasectomy remain at substantially elevated risk of death despite resection with curative intent, and their recurrence is frequently extrahepatic and early. This argues for intensified post-operative surveillance in this group, for consideration of adjuvant systemic strategies, and for prioritisation in trials of perioperative therapy. As immunotherapy combinations and novel targeted agents (e.g., BRAF- and KRAS G12C-directed regimens) reshape treatment even in traditionally resistant subgroups [28,29,30,31,32], their interaction with surgical strategy will grow more complex, reinforcing the need for a unified, biology-driven approach.

Several limitations warrant consideration. The retrospective design carries inherent risks of selection bias and unmeasured confounding, particularly in the assessment of surgical benefit. The absence of comprehensive molecular profiling—including BRAF status and microsatellite instability—limits full biological characterisation, and the multi-era span introduces heterogeneity in systemic regimens. Subgroup analyses within the metastasectomy cohort are limited by sample size, yielding wide confidence intervals; the corresponding estimates should be read as hypothesis generating. Nonetheless, the consistency of our findings with established biological principles and contemporary trial evidence supports their robustness.

Ultimately, these data reinforce a single principle: in metastatic colorectal cancer, anatomy defines the boundaries of intervention, but biology defines the limits of control. Any surgical strategy that fails to incorporate this principle risks misalignment between therapeutic intent and clinical outcome [33,34,35].

## 5. Conclusions

In this real-world mCRC cohort, metastasectomy was associated with a substantial survival benefit that was neither uniform nor independent of tumour biology. RAS mutational status and primary tumour sidedness emerged as independent prognostic determinants, including among surgically resected patients, in whom five-year survival differed by thirty-five absolute percentage points according to genotype alone; right-sided primary tumour location carried a significant survival disadvantage that was largely accounted for by its correlation with RAS status.

These findings highlight the limitations of anatomy-based surgical paradigms and support integrating molecular and clinicopathological factors into the planning of care after resection—through intensified surveillance, adjuvant strategies and trial enrolment—rather than into the decision to operate. A biologically informed approach to post-operative management may better align therapeutic strategy with long-term disease control.

## Figures and Tables

**Figure 1:**
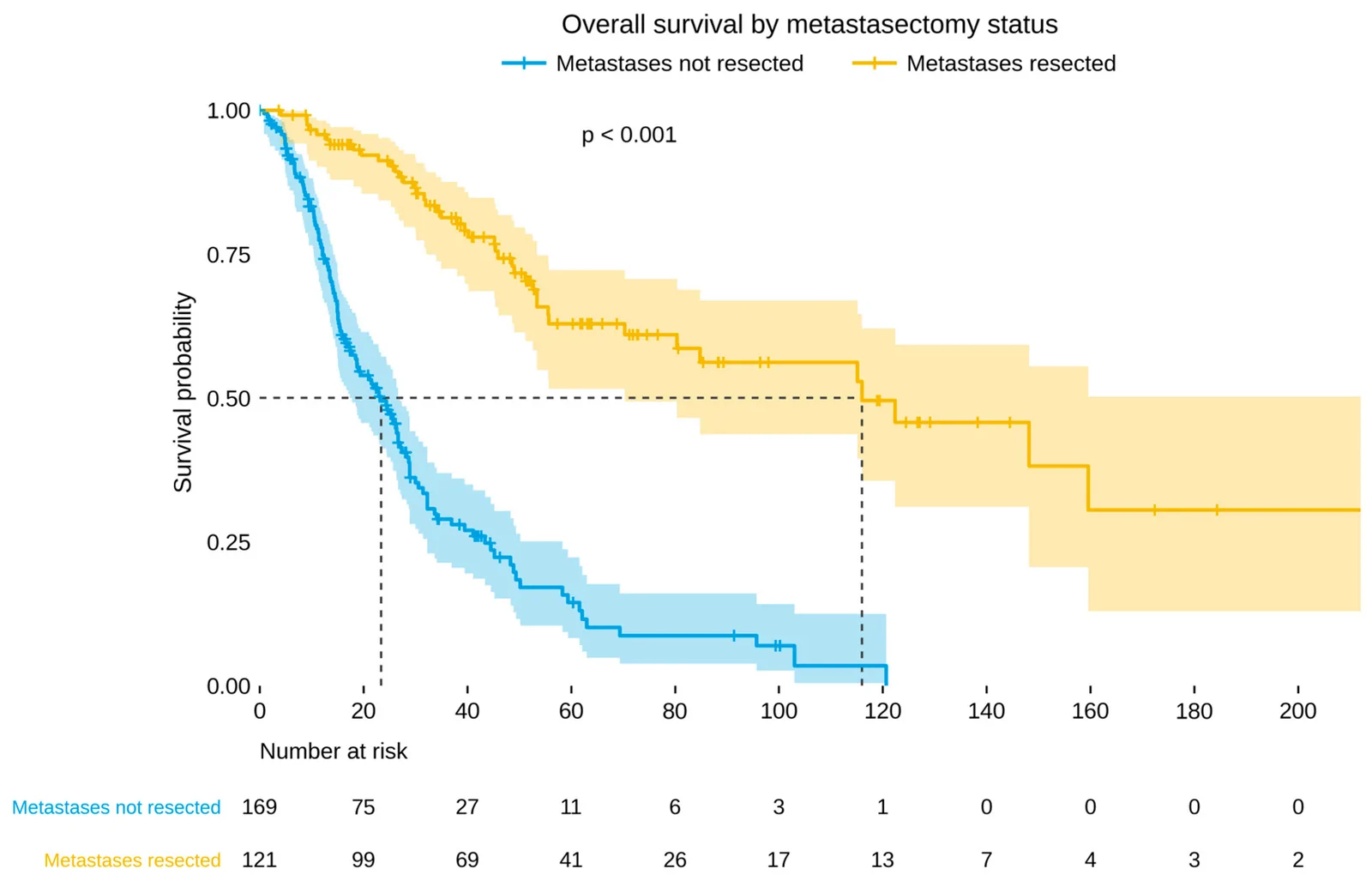
Kaplan–Meier curve for overall survival by metastasectomy status.

**Figure 2:**
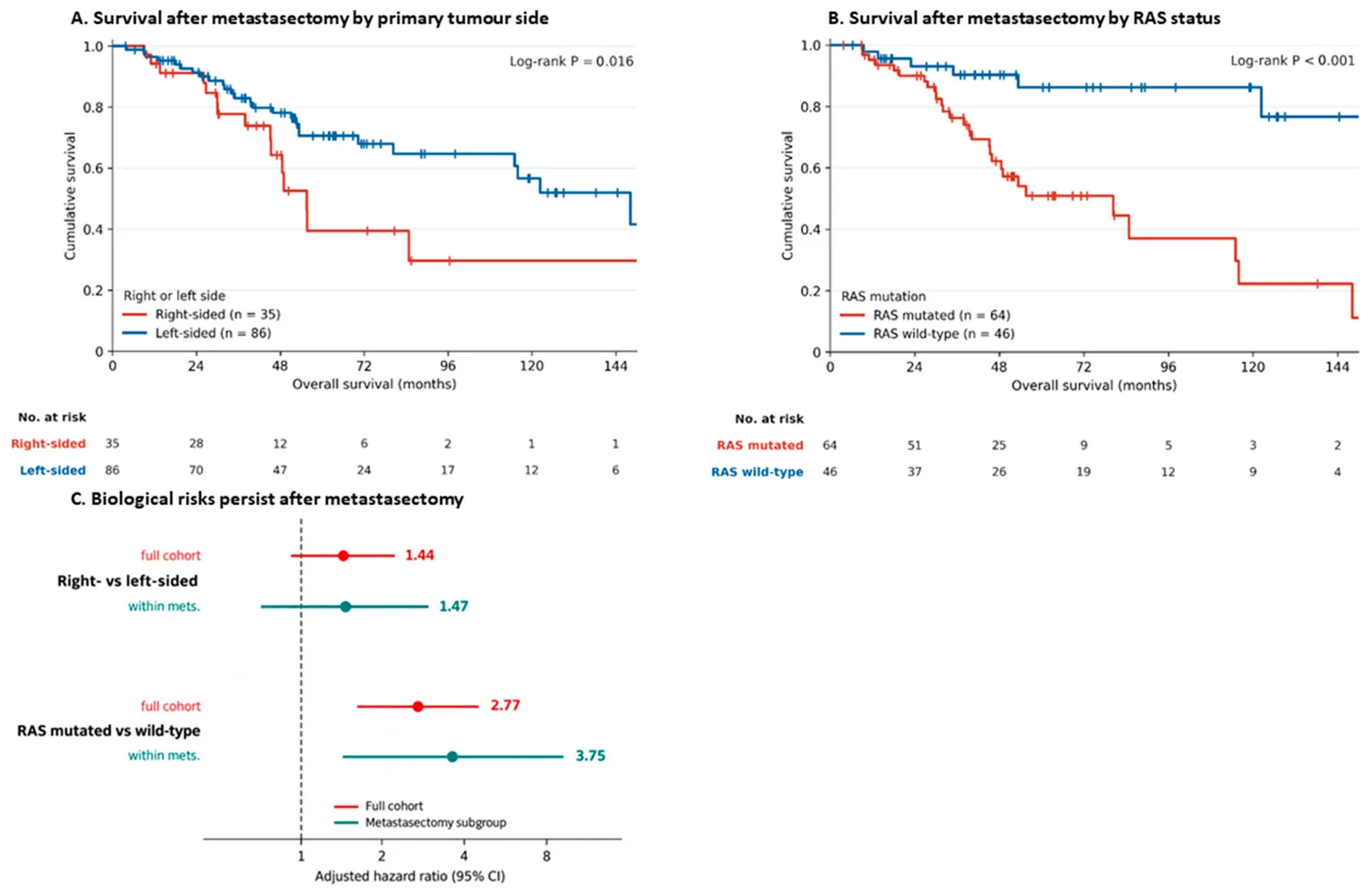
Tumour biology persists beyond resectability. (**A**) Kaplan–Meier survival curve after metastasectomy by primary tumour side. (**B**) Kaplan–Meier survival curve after metastasectomy by RAS mutation. (**C**) Hazard ratio by side and RAS mutated in the full cohort and metastasectomy subgroup.

**Table 1:** Baseline characteristics of the study cohort (n = 293), overall and according to metastasectomy status.

Variable	All Patients (n = 293)	Metastasectomy (n = 121)	No Metastasectomy (n = 169)	*p* Value
**Sex**				0.290
Female	134 (45.7)	60 (49.6)	72 (42.6)	
Male	159 (54.3)	61 (50.4)	97 (57.4)	
**Age, years**				0.001
Median (range)	62 (23–98)	58 (23–84)	64 (34–98)	
Mean ± SD	61.4 ± 13.38	58.1 ± 13.46	63.8 ± 13.53	
**ECOG performance status**				0.001
0	204 (69.6)	96 (79.3)	107 (63.3)	
1	75 (25.6)	25 (20.7)	49 (29.0)	
≥2	14 (4.7)	0 (0.0)	13 (7.7)	
Comorbidity *				0.117
No	139 (48.9)	66 (54.5)	71 (44.4)	
Yes	145 (51.1)	55 (45.5)	89 (55.6)	
Histology *				0.156
Adenocarcinoma	272 (92.8)	108 (89.3)	17 (77.3)	
Mucinous	21 (7.2)	13 (10.7)	5 (22.7)	
Differentiation *				0.026
Well	17 (6.2)	13 (10.7)	2 (1.9)	
Moderate	219 (79.3)	94 (77.7)	92 (86.0)	
Poor	40 (14.5)	14 (11.6)	13 (12.1)	
**Pathological T stage †**				0.323
pT1–2	17 (12.2)	14 (11.6)	3 (15.8)	
pT3	99 (70.7)	84 (69.4)	15 (78.9)	
pT4	24 (17.1)	23 (19.0)	1 (5.3)	
**Pathological N stage †**				0.847
pN0	36 (25.7)	32 (26.4)	4 (21.1)	
pN1	66 (47.1)	56 (46.3)	10 (52.6)	
pN2	38 (27.1)	33 (27.3)	5 (26.3)	
**Primary tumour location ‡**				0.145
Right colon	99 (33.6)	35 (28.9)	64 (37.9)	
Left-sided group	194 (66.4)	86 (71.1)	105 (62.1)	
**RAS status §**				0.166
Mutated	138 (63.6)	64 (58.2)	71 (68.3)	
Wild-type	79 (36.4)	46 (41.8)	33 (31.7)	
**Metastatic presentation**				<0.001
Synchronous	163 (55.6)	51 (42.1)	110 (65.1)	
Metachronous	130 (44.4)	70 (57.9)	59 (34.9)	
**Metastasectomy**				
No	172 (58.7)	—	169 (100)	
Yes	121 (41.3)	121 (100)	—	
Site of metastatic disease at presentation *				<0.001
Liver only	107 (42.1)	43 (50.6)	64 (37.9)	
Lung only	39 (15.4)	19 (22.4)	20 (11.8)	
Liver and lung	26 (10.2)	4 (4.7)	22 (13.0)	
Intra-abdominal/peritoneal only	22 (8.7)	12 (14.1)	10 (5.9)	
Other single site (ovary, adrenal, locoregional)	6 (2.4)	5 (5.9)	1 (0.6)	
Other or multiple sites	54 (21.3)	2 (2.4)	52 (30.8)	
**Site of resected metastatic disease (n = 121) ‖**				
Liver		65 (53.7)	—	
Lung		23 (19.0)	—	
Liver and lung		9 (7.4)	—	
Peritoneum		13 (10.7)	—	
Other sites (regional lymph nodes, ovary, local recurrence)		11 (9.1)	—	
**Resection margin status (n = 121) ¶**				
R0		90 (74.4)	—	
R1		1 (0.8)	—	
Not recorded		30 (24.8)	—	

* Values are n (%) unless otherwise stated. The ‘all patients’ column reproduces the originally submitted Table 1 (n = 293). Group columns were derived from the individual patient records available for the survival analyses (n = 290; three patients of the non-operative group lacked an individual record), which explains minor differences between the sum of the group columns and the overall column. *p* values compare the metastasectomy and non-operative groups: chi-square or Fisher’s exact test for categorical variables; Mann–Whitney U test for age. Percentages from patients with available data. † Available for the subgroup undergoing primary tumour resection. ‡ The dataset grouped descending/sigmoid/rectal tumours in the left-sided comparison for survival analyses. § RAS status available only for tested patients. ‖ Site of resected metastatic disease as reported in the source cohort (n = 121). ¶ Resection margin status from individual records; not recorded in 30 patients.

**Table 2:** Multivariable Cox regression for overall survival—full cohort.

Variable	Comparison	HR (95% CI)	*p*
Age	<70 vs. ≥70	0.58 (0.37–0.92)	0.021
Primary tumour location	Right vs. left	1.44 (0.93–2.23)	0.100
Metastasectomy	No vs. yes	4.58 (2.82–7.44)	<0.001
Metastatic presentation	Metachronous vs. synchronous	0.61 (0.39–0.95)	0.028
RAS status	Mutated vs. wild-type	2.77 (1.65–4.64)	<0.001

Subgroup restricted to patients who underwent resection of metastatic disease with curative intent; n = 110, 35 events. Unadjusted estimates within this subgroup are 5.52 (95% CI 2.25–13.51; *p* < 0.001) for RAS mutation and 2.15 (1.14–4.05; *p* = 0.018) for right-sided location. The number of covariates was constrained by the number of events observed.

**Table 3:** Multivariable Cox regression in patients undergoing metastasectomy.

Variable	Comparison	HR (95% CI)	*p*
Age	<70 vs. ≥70	0.47 (0.21–1.09)	0.079
Primary tumour location	Right vs. left	1.47 (0.72–2.99)	0.292
Pathological nodal status	N0–1 vs. N2	0.38 (0.18–0.77)	0.008
RAS status	Mutated vs. wild-type	3.75 (1.46–9.60)	0.006

## Data Availability

The original contributions presented in this study are included in the article. Further inquiries can be directed to the corresponding author.

## Supplementary Materials

The following supporting information can be downloaded at https://www.mdpi.com/article/10.3390/cancers18193070/s1: Table S1: Sensitivity analyses addressing the timing of metastasectomy and of systemic therapy in the full cohort.
