# Supplementary material for: Integrating Tumour Biology into Surgical Decision-Making for Metastatic Colorectal Cancer: A Real-World Cohort Analysis Adapted for Clinical Colorectal Cancer
Source: Cancers (Basel). 2026 Sep 22;18(19):3070. doi: 10.3390/cancers18193070 (PMC13664121; doi:10.3390/cancers18193070)
Supplement: Supplementary file 1 [file cancers-18-03070-s001.zip › cancers-4529924-supplementary.pdf]

Supplemental Table S1. Sensitivity analyses addressing the timing of metastasectomy and of systemic therapy in the full cohort

**Specification**	**HR (95% CI)**	**P**
No metastasectomy, baseline covariate	4.58 (2.82–7.44)	< 0.001
No metastasectomy, time-dependent covariate	3.54 (2.17–5.77)	< 0.001
No metastasectomy, landmark at 3 months	2.30 (1.39–3.81)	0.001
No metastasectomy, landmark at 6 months	2.50 (1.53–4.09)	< 0.001
No metastasectomy, landmark at 12 months	3.14 (1.87–5.26)	< 0.001
Incomplete systemic therapy, time-dependent	1.25 (0.81–1.92)	0.323

The median interval from diagnosis of metastatic disease to surgery was 216 days (interquartile range 85–323). HR: Hazard Ratio. CI: Confidence Interval.
